# Multivariate Spatial Condition Mapping Using Subtractive Fuzzy Cluster Means

**DOI:** 10.3390/s141018960

**Published:** 2014-10-13

**Authors:** Hakilo Sabit, Adnan Al-Anbuky

**Affiliations:** Electrical and Electronic Engineering, Auckland University of Technology, 24 St Paul Street, Auckland 1010, New Zealand; E-Mail: aalanbuk@aut.ac.nz

**Keywords:** data stream mining, sensor cloud, fuzzy clustering, wireless sensor network

## Abstract

Wireless sensor networks are usually deployed for monitoring given physical phenomena taking place in a specific space and over a specific duration of time. The spatio-temporal distribution of these phenomena often correlates to certain physical events. To appropriately characterise these events-phenomena relationships over a given space for a given time frame, we require continuous monitoring of the conditions. WSNs are perfectly suited for these tasks, due to their inherent robustness. This paper presents a subtractive fuzzy cluster means algorithm and its application in data stream mining for wireless sensor systems over a cloud-computing-like architecture, which we call sensor cloud data stream mining. Benchmarking on standard mining algorithms, the k-means and the FCM algorithms, we have demonstrated that the subtractive fuzzy cluster means model can perform high quality distributed data stream mining tasks comparable to centralised data stream mining.

## 1. Introduction

Wireless sensor networks (WSNs) are usually deployed to measure given physical phenomena over a certain space, within a specific time frame. The spatio-temporal distribution of these phenomena often correlates with certain physical events. To appropriately characterise these events-phenomena relationships over a given space for a specific time frame, continuous monitoring of the conditions is required. WSNs are perfectly suited for these tasks due to their inherent robustness. For instance, spatio-temporal distribution of weather conditions over a forestry area closely correlate with the likelihood of forest fire events in the area. WSNs can be deployed to continuously measure the weather conditions at very precise time and space-scales. WSNs can feed the current levels of these conditions into forest fire event monitoring and prediction algorithms. As a result, the computation of the likelihood of these events can be automated and graphically presented. This problem falls under the general category of multivariate spatial condition mapping, with the added complexity of continuously streaming data.

The physical sensor cloud's ability to run the computation of a given task distributed over a number of wirelessly connected nodes in parallel over a coordinated network platform resembles the cloud computing architecture. In this paper, we propose a subtractive fuzzy cluster means algorithm and its application in data stream mining for wireless sensor systems over a physical cloud-computing-like architecture, which we call sensor cloud data stream mining.

The motivational example for our paper arises from wildfire event mapping experiences. Wildfires play an important role in the structure and functioning of many of of the world's ecosystems [1]. The wildfire activity patterns that generally characterise a given area change with alterations in temperature and precipitation conditions under climate change [2,3]. Therefore, there is a need to characterise the complex relationship between wildfires, climate, vegetation and human activities at fine scales.

**Figure 1.**
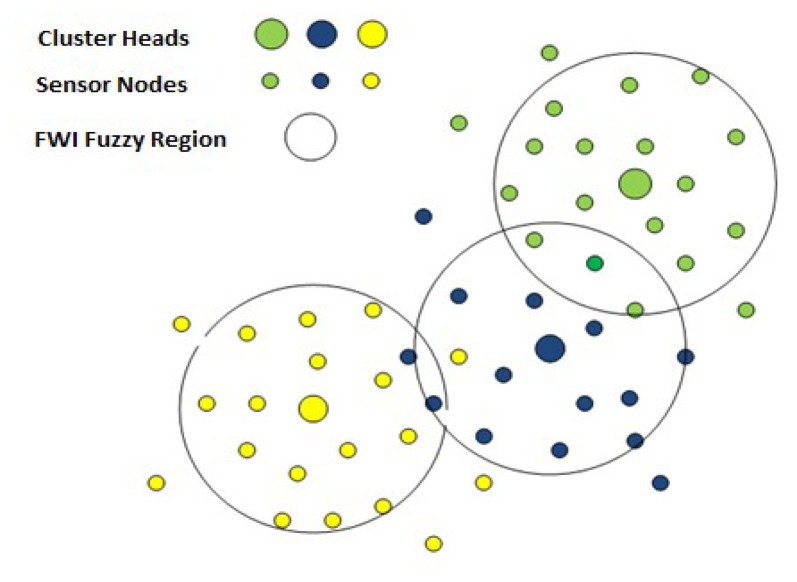
Spatial map of weather variables and the fire weather index (FWI).

The Canadian Fire Weather Index (FWI) system is an example of such a system. The FWI system is an estimation of the risk of wildfire based on the empirical model developed by Van Wagner [4]. FWI is used to estimate fuel moisture content and generate a series of relative fire behaviour indices based on weather observations. The fuel moisture and fire behaviour indices are used by operational personnel to aid in the estimation of expected daily fire occurrence, potential fire behaviour and the difficulty of suppression across a fire management district, region or province [5]. The complex relationship between forest weather variables and the forest floor moisture profiles is modelled by the FWI system. The system represents the forest floor moisture profile by a set of codes and indexes known as fuel moisture codes and fire behaviour indexes. We use the FWI indexes for producing spatial maps of wildfire events, as shown in Figure 1.

### 1.1. Spatial Condition Clustering

Given a set of data, clustering is the method of categorizing data into groups based on similarity. It has been used in exploratory data analysis to find unexpected patterns in datasets. It is also referred to as unsupervised learning or data mining. Clustering is inherently an ill-defined problem. Input data to the clustering algorithm is usually a vector (also known as “tuple” or “record”), which may be numerical, categorical or Boolean. Clustering algorithms must include a method for computing the similarity of or distance between vectors. Distance is the most natural method for numerical data similarity measurement. Lower values indicate more similarity. Common distance metrics include Euclidean distance and Manhattan distance. However, the distance metric does not generalise well to non-numerical data. The detailed description of basic clustering concepts, including major phases involved in clustering a dataset and examples from various areas, including biology, healthcare, market research, *etc.* can be found in [6]. k-means and fuzzy c-means (FCM) clustering algorithms have been two of the most popular algorithms in this field.

k-means [7] is one of the simplest unsupervised learning algorithms that solves the well-known clustering problem. Given a set of numeric points in d-dimensional space and an integer, the k-means algorithm generates k or fewer clusters by assigning all points to a cluster at random and repetitively computes the centroid for each cluster, reassigning each point to the nearest centroid until all clusters are stable. The major limitation of k-means is that the parameter k must be chosen in advance.

Fuzzy c-means (FCM) is a method of clustering developed by Dunn [8] and later improved by Bezdek [9]. FCM allows a piece of data to belong to two or more clusters with varying degrees of membership. In real applications, there is often no sharp boundary present between neighbouring clusters. This leads to fuzzy clustering being better suited to the data. Membership degrees between zero and one are used in fuzzy clustering instead of crisp assignments of the data to clusters. The most prominent fuzzy clustering algorithm is the fuzzy c-means, a fuzzification of k-means. A detailed description of the fundamentals of fuzzy clustering, basic algorithms and their various realisations, as well as cluster validity assessment and result visualisation are provided in [10]. Similar to k-means, a major drawback of FCM in exploratory data analysis is that it requires the number of clusters within the data space to be known beforehand. When the purpose of clustering is to automatically partition multivariate data coming from a dynamic source, the number of partitions in the data space is typically unknown. Hence, in this research, subtractive clustering and FCM algorithms are combined to implement an algorithm that does not require prior information of the number of clusters in the data space. The proposed algorithm is called the subtractive fuzzy cluster means algorithm (SUBFCM) [11].

### 1.2. Data Stream Mining Algorithm

Data stream mining is the process of extracting knowledge structures from continuous data streams. A data stream is an ordered sequence of instances that in many applications of data stream mining can only be read a few times using limited computing and storage capabilities. Examples of data streams include among others, computer network traffic, phone conversations, ATM transactions, web searches and sensor data. In data mining, we are interested in techniques to find and describe structural patterns in the data, as a tool to help explain the data and make predictions from it [12]. One of the popular data mining techniques in a centralised environment is data clustering. The general goal of the clustering technique is to decompose or partition datasets into groups, such that both intra-group similarity and inter-group dissimilarity are maximised [13]. WSNs can benefit a great deal from stream mining algorithms in terms of energy conservation and efficient services. However, for WSNs to achieve significant energy conservation, the data stream mining has to be performed distributed within the network, due to their resource constraints [11,14]. In a distributed (non-centralised) computing environment, the most prominent works include the state-of-the-art in learning from data streams [15], dynamic learning models in non-stationary environments [16] and incremental or evolving clustering methods [17–19]. Data mining applications place special requirements on clustering algorithms available to the WSN nodes, including the ability to find clusters embedded in subspaces of high dimensional data and scalability. The algorithm is also required to produce frequent summaries of the corresponding inputs from the network sensor nodes. In stream mining [20,21], WSN data mining applications further place strict requirements on the underlying algorithm. Collecting data generated in a WSN to a central location and performing data mining is undesirable, due to the energy and bandwidth limitations. Therefore, the data mining algorithm has to perform in-network and autonomously on limited resource applications. The algorithm has to converge as fast as possible over the limited datasets to ensure that the processor can take on the next set of streams. The novelties of this work lie in concurrently addressing these WSN requirements, namely in-network computing, autonomously deciding the number of cluster centres, limited utilisation and high rate of convergence.

### 1.3. Subtractive Clustering Method

The subtractive clustering method was developed by Chiu [22]. It is a modification of mountain clustering [23] with improved computational complexity. This clustering method assumes that each data point is a potential cluster centre (prototype). A data point with more neighbouring data will have a higher potential to become a cluster centre than points with fewer neighbouring data. In subtractive clustering method, the computation is proportional to the number of data points and is independent of the dimension of the problem. Subtractive clustering considers a data point with the highest potential as a cluster centre and penalises data points close to the new cluster centre to facilitate the emergence of new cluster centres. Based on the density of surrounding data points, the potential value for each data point is calculated as follows:

(1) $${\text{pot}}_{i}=\sum_{j=1}^{m}e^{-\alpha {\left\|u_{i}-u_{j}\right\|}^{2}}$$

where *u_i_*, *u_j_* are data points and $\alpha =\frac{4}{r_{a}^{2}}$ and *r_a_* is a positive constant defining a neighbourhood. Data points outside this range have little influence on the potential. Following the potential calculation of every data point, the point with the highest potential is chosen as the first cluster centre. Let *u_k_* be the location of the first cluster centre and *pot_k_* be its potential value. The potential of the remaining data points *u_i_* is then revised by:

(2) $${\text{pot}}_{i}={\text{pot}}_{i}-{\text{pot}}_{k}e^{-\beta \|u_{i}-u_{k}{|}^{2}}$$

where $\beta =\frac{4}{r_{b}^{2}}$ and *r_b_* is a positive constant (*r_b_* > *r_a_*).

Thus, the data points near the first cluster centre will have greatly reduced potential and, therefore, are unlikely to be selected as the next cluster centre. The constant *r_b_* is the neighbourhood defining radius and will have a significant reduction in potential. *r_b_* is set to be greater than *r_a_* to avoid closely-spaced centres. The ratio between *r_a_* and *r_b_* is called the squash factor (SF), which is a positive constant greater than one. The subtractive clustering algorithm is shown in Algorithm 1.


**Algorithm 1.** Subtractive clustering algorithm.
Inputs: {*u*_1_, *u*_2_, *u*_3_, …*u_i_*} Outputs: {*c*_1_, *c*_2_, *c*_3_, …*c_C_*}Initialise: *r_a_*, *r_b_*, *AR*, *RR*
1.Calculate potential of each data point, Equation (1)2.Set the maximum potential as *pot_k_*3.choose the the data point corresponding to *pot_k_* as a cluster centre candidate4.If *pot_i_* > *AR* * *pot_k_*, then accept *u_i_* as a cluster centre5. Update the potential of each point using Equation (2) and continue6. Else if *pot_i_* < *RR* * *pot_k_*, then reject *u_i_* as a cluster centre7.Else8. Let *d_r_* be relative distance9. If 
$\frac{d_{r}}{r_{a}}+\frac{{\text{pot}}_{k}}{{\text{pot}}_{i}}\geq 1$ accept *u_i_* as a cluster centre10.  Update the potential of each point using Equation (2) and continue11.  else12.  reject *u_i_* and set the potential *pot_i_* = 013.  Select the data point with the next highest potential as the new candidate and re-test14. end if15.end if


The potential update process (2) will continue until no further cluster centre is found. The parameters known as the accept ratio (AR) and reject ratio (RR) together with the influence range and squash factor set the criteria for the selection of cluster centres. The accept ratio and reject ratio are the upper acceptance threshold and lower rejection threshold, respectively, and they take a value between zero and one. The accept ratio should be greater than the reject ratio.

First criteria: If the potential value ratio of the current data point to the original first cluster centre is larger than the accept ratio, then the current data point is chosen as a cluster centre.

Second criteria: If the potential value falls in between that of the accept and reject ratios, then the compensation between the magnitude of that potential value and the distance from this point to all of the previous chosen cluster centres (relative distance) is taken into consideration. If the sum of the potential value and the ratio of the shortest distance between the current data point and all other previously found cluster centres to the influence range is greater than or equal to one, then the current data point is accepted as a cluster centre.

Third criteria: If the sum of the potential value and the ratio of the shortest distance between the current data point and all other previously found cluster centres to the influence range is less than one, then the current data point is rejected as a cluster centre.

Forth criteria: If the potential value ratio of the current data point to the original first cluster centre is less than the reject ratio, then the potential value of the current data point is revised to zero and the data point with the next highest potential is tested.

### 1.4. Fuzzy C-Means Clustering

Fuzzy clustering algorithms are based on minimisation of the fuzzy c-means objective function formulated as:

(3) $$J_{o}=\sum_{c=1}^{C}\sum_{i=1}^{m}{\left(\upsilon_{ci}\right)}^{\theta }{\left\|u_{i}-\upsilon_{i}\right\|}^{2}A$$

where *υ_ci_* is a fuzzy partition matrix of *u*,

(4) $$\upsilon =[\upsilon_{1},\upsilon_{2},\upsilon_{3},\ldots \upsilon_{c}]$$

is a vector of cluster centres, which have to be determined,

(5) $$d_{ciA}^{2}={\left\|u_{i}-\upsilon_{i}\right\|}_{A}^{2}={\left(u_{i}-\upsilon_{i}\right)}^{T}A(u_{i}-\upsilon_{i})$$

is a squared inner-product distance norm and

(6) θ∈[1,∞]

is a parameter that determines the fuzziness of the resulting clusters.

The conditions for a fuzzy partition matrix are given as:

(7) $$\begin{array}{ccc} \upsilon_{ci}\in [0,1], & 1\leq c\leq i, & 1\leq i\leq n \end{array}$$

(8) $$\begin{array}{cc} \overset{c}{\underset{i=1}{\text{∑}}}\upsilon_{ci}=1, & 1\leq i\leq n \end{array}$$

(9) $$\begin{array}{cc} 0<\overset{n}{\underset{i=1}{\text{∑}}}\upsilon_{ci}<N, & 1\leq i\leq c \end{array}$$

The value of the objective function (3) can be seen as a measure of the total variance of *u_i_* from *υ_i_*. The minimisation of the objective function (3) is a non-linear optimisation problem that can be solved by iterative minimisation, simulated annealing or genetic algorithm methods. The simple iteration method through the first-order conditions for stationary points of (3) is known as the fuzzy c-means (FCM) algorithm.

The stationary points of the objective function (3) can be found by adjoining the constraint (8) to *J_o_* by means of Lagrange multipliers:

(10) $$J=\sum_{c=1}^{C}\sum_{i=1}^{m}{\left(\upsilon_{ci}\right)}^{\theta }d_{ciA}^{2}+\sum_{i=1}^{n}\lambda_{i}\left[\sum_{i=1}^{c}\upsilon_{ci}-1\right]$$

and by setting the gradient of *J* with respect to the fuzzy partition matrix *u*, the vector of cluster matrix *υ* and λ to zero.

Now, if $d_{ciA}^{2}>0$, λ*_i_*, *c* and *θ* > 1, then (*u*, *υ*) may minimise the objective function (3) only if:

(11) $$\begin{array}{ccc} \upsilon_{ci}=\frac{1}{\overset{c}{\underset{j=1}{\text{∑}}}{\left(d_{ciA}/d_{cjA}\right)}^{2}/\left(\theta -1\right)}, & 1\leq j\leq c, & 1\leq i\leq n \end{array}$$

and:

(12) $$\begin{array}{cc} \upsilon_{i}=\frac{\overset{n}{\underset{i=1}{\text{∑}}}{\left(\upsilon_{ci}\right)}^{\theta }u_{i}}{\overset{n}{\underset{i=1}{\text{∑}}}{\left(\upsilon_{ci}\right)}^{\theta }}; & 1\leq i\leq c \end{array}$$

This solution also satisfies the Equations (7) and (9). Equations (11) and (12) are the first-order necessary conditions for stationary points of the objective function (3). The FCM algorithm iterates through Equations (11) and (12). The sufficiency of the necessary Equations (11) and (12), as well as the convergence of the FCM algorithm is proven in [24].

Before using the FCM algorithm, the parameters are: the number of clusters, *C*, the fuzziness exponent, *θ*, the termination tolerance, *ϵ*, and the norm-inducing matrix, *A*. The fuzzy partition matrix, *u*, must also be initialised. Note that the FCM algorithm converges to a local minimum of the objective function (3); hence, different initialisations may lead to different results. The Fuzzy c-means algorithm is shown in Algorithm 2.


**Algorithm 2.** Fuzzy c-means clustering algorithm.
**Inputs:** {*c*_1_, *c*_2_, *c*_3_,…*c_C_*} {*u*_1_, *u*_2_, *u*_3_, …*u_i_*}**Outputs:**
*υ_i_*, *i* = 1, 2, 3, …*C U_i,j_*, *i* = 1, 2, ..*C*, *j* = 1, 2, …*n***Initialise:**
*ϵ*, *θ, A*
1.For, *l* = 1, 2, 3, … Repeat2.Compute cluster centres (prototypes):3.
$\upsilon_{i}=\frac{\overset{n}{\underset{i=1}{\text{∑}}}{\left(\upsilon_{ci}\right)}^{\theta }u_{i}}{\overset{n}{\underset{i=1}{\text{∑}}}{\left(\upsilon_{ci}\right)}^{\theta }}$; 1 ≤ *i* ≤ *c*4.Compute distances:5.
$d_{ciA}^{2}={\left(u_{i}-\upsilon_{i}\right)}^{T}A(u_{i}-\upsilon_{i})$, 1 ≤ *c* ≤ *C*, 1 ≤ *i* ≤ *n*6.Update the partition matrix:7.For 1 ≤ *i* ≤ *n*8. If *d_ciA_* > 0 for all *c* = 1, 2, …, *C*9.  
$\upsilon_{ic}=\frac{1}{\sum_{j=1}^{C}{\left(d_{ciA}/d_{cjA}\right)}^{2}/\left(\theta -1\right)}$10. Else11.  *υ_ci_* = 0 if *d_ci_* > 0 and *υ_ci_* ∋ [0, 1] with 
$\overset{C}{\underset{i=1}{\text{∑}}}\upsilon_{ci}=1$12.  until 
$\left\|\upsilon_{ci}^{\left(l\right)}-\upsilon_{ci}^{\left(l-1\right)}\right\|<\epsilon$


### 1.5. The SUBFCM Algorithm

Embedding an autonomous cluster mining algorithm in WSN nodes requires that the algorithm take datasets as input and generates output without data preprocessing. In applications where the number of clusters in a dataset must be discovered, the FCM algorithm cannot be used directly. For clustering WSN data autonomously, the number of cluster prototypes (categories) has to be determined from the datasets. Hence, in this research, subtractive clustering and FCM algorithms are combined to implement an algorithm that determines the number of clusters in the data space from the input datasets: subtractive fuzzy cluster means (SUBFCM).

The SUBFCM algorithm uses a subtractive clustering approach to determine the number of cluster prototypes *C* and the prototype centres *c*. The algorithm then partitions the stream into *C* fuzzy clusters using the prototype centres from the above step as initial fuzzy cluster centres.

Initially, the SUBFCM algorithm assumes each D-dimensional data point *u_i_*, *i* = 1, 2, 3, …, *m* as a potential cluster centre with a measure of the potential (*pot*) of data points in the stream as:

(13) $${\text{pot}}_{i}=\sum_{j=1}^{m}e^{-\alpha {\left\|u_{i}-u_{j}\right\|}^{2}}$$

where $\alpha =\frac{4}{r_{a}^{2}}$ and *r_a_* is a positive constant defining the cluster radius. A large value of *r_a_* results in fewer large clusters, while smaller values result in more smaller diameter clusters. ‖ ‖ Denotes the Euclidean distance, which defines the distance between two points *u_i_*(*x*_1_, *y*_1_, *z*_1_) and *u*_2_(*x*_2_, *y*_2_, *z*_2_) as being equal to the length of the vector.

(14) $$\left\|X_{1}-X_{2}\right\|=\sqrt{{\left(x_{1}-x_{2}\right)}^{2}+{\left(y_{1}-y_{2}\right)}^{2}+{\left(z_{1}-z_{2}\right)}^{2}}$$

where

$$X_{1}\equiv \left[\begin{array}{c} x_{1}\\ y_{1}\\ z_{1} \end{array}\right]$$

and

$$X_{2}\equiv \left[\begin{array}{c} x_{2}\\ y_{2}\\ z_{2} \end{array}\right]$$

The measure of potential for a given data point is a function of its distances to all other points. A data point with many neighbouring points will have a high potential value. After computing the potential for every point, the point *u_k_* with the highest potential *pot_k_* will be selected as the first cluster centre *c*_1_. The potential for every other point is then updated by Equation (15):

(15) $${\text{pot}}_{i}={\text{pot}}_{i}-{\text{pot}}_{k}e^{-\beta {\left\|u_{i}-u_{k}\right\|}^{2}}$$

where $\beta =\frac{4}{r_{b}^{2}}$ and *r_b_* is a positive constant that can be set to a value that is greater than *r_a_*. After the first cluster centre is determined, the value of *r_b_* determines the potential of data points becoming subsequent cluster centres. Setting *r_b_* > *r_a_* reduces the potential of data points close to the first cluster centre and, hence, avoids closely-spaced cluster centres [25]. The parameter *α* is a stopping criterion and should be selected within (0, 1) [26]. *α* with a value close to zero will result in a large number of hidden centres, whereas *α* close to one leads to a small network structure.

Following the update process, the data point with the highest remaining potential is selected as the next cluster centre *c*_2_, and the process repeats until a given threshold *ε* for the potential is reached and *C* such centres are computed. SUBFCM then uses the clustering criterion of squared distance $d_{ci}^{2}$ between the *i* – *th* stream sample and the *c* – *th* prototype and defines the objective function (*J*_0_) as:

(16) $$J_{0}=\sum_{c=1}^{C}\sum_{i=1}^{m}\upsilon_{ci}^{\theta }d_{ci}^{2}$$

where the squared distance function is given as:

(17) $$d_{ci}^{2}={\left\|u_{i}-Cc\right\|}^{2}$$

where *υ_ci_* represents the membership degree of the *i* – *th* stream sample to the *c* – *th* cluster.

Membership is determined under the conditions:

(18) $$\begin{array}{ccc} \upsilon_{ci}\in [0,1], & c=1,2,3,\ldots ,C & i=1,2,3,\ldots m \end{array}$$

and:

(19) $$\begin{array}{cc} \overset{C}{\underset{c=1}{\text{∑}}}\upsilon_{ci}=1, & i=1,2,3,\ldots ,m \end{array}$$

where *θ* is a weighing exponent or fuzziness measure. If *θ* = 1, the clustering model is reduced to the hard k-means model. The larger the *θ*, the fuzzier the memberships is. *θ* is usually set to two [24].

The stream partitioning takes place by optimizing the criterion function (16) through iteration, updating the cluster prototype centres *c_j_* and the membership function *υ_ci_* as Equations (20) and (21) respectively:

(20) $$c_{j}=\frac{\overset{m}{\underset{i=1}{\text{∑}}}{\left(\upsilon_{ij}\right)}^{\theta }u_{i}}{\overset{m}{\underset{i=1}{\text{∑}}}{\left(\upsilon_{ij}\right)}^{\theta }}$$

(21) $$\upsilon_{ci}={\left(\sum_{l=1}^{C}{\left(\frac{\left\|u_{i}-c_{j}\right\|}{\left\|u_{i}-c_{l}\right\|}\right)}^{2/(\theta -1)}\right)}^{-1}$$

The iteration should stop when:

(22) $$\max=\left\{\left|\upsilon_{ci}^{\left(l+1\right)}-\upsilon_{ci}^{\left(i\right)}\right|\right\}<\epsilon$$

where *ϵ* is the termination criterion, 0 < *ϵ* < 1 and *l* is the iteration step.

SUBFCM takes the fuzzy radius *r_a_* and fuzziness measure *θ* as inputs and autonomously reveals the structures in the data stream space. The parameter *r_a_* determines the granularity of the structures. The smaller it is, the higher the resolution of the structures and the more computational overhead. The SUBFCM algorithm is shown in Algorithm 3.


**Algorithm 3.** The subtractive fuzzy cluster means (SUBFCM) algorithm.
Inputs: {*u*_1_, *u*_2_, *u*_3_, …*u_i_*}Outputs: *υ_i_*, *i* = 1, 2, 3, …*C U_i,j_*, *i* = 1, 2, ..*C*, *j* = 1, 2, …*n*Initialise: *ϵ*, *θ*, *r_a_*, *α*
1.For, *i* = 1 ← *to m* Repeat2.Compute potential (*pot_i_*) using Equation (13):3.Set *c_j_* = *pot_k_*4.Do5. For *i* = 1 ← *to m* − 16.  Compute *pot_i_* using Equation (15)7.  Set *c_j_*_+1_ = *pot_k_*8.while *pot_i_* > *α*9.Do10. For, *i* = 1 ← *to m*11.  Compute *d_ci_* using Equation (19)12.  Set *υ_ci_* = *d_ci_*13.  Compute *c_j_* using Equation (22)14.  Compute *υ_ci_* using Equation (23)15.while 
$\upsilon_{ci}^{\left(l+1\right)}-\upsilon_{ci}^{\left(l\right)}>\epsilon$


## 2. Simulation and Analysis

Both the SUBFCM algorithm and the target application performances are evaluated based on simulations using TrueTime, a MATLAB/Simulink-based simulator for real-time control network systems. TrueTime facilitates co-simulation of task execution in real-time kernels, network transmissions and continuous system dynamics. The TrueTime kernel block and wireless network blocks parameters are tuned for a small-scale physical WSN deployment.

### 2.1. SUBFCM Performance Characterisation

**Figure 2.**
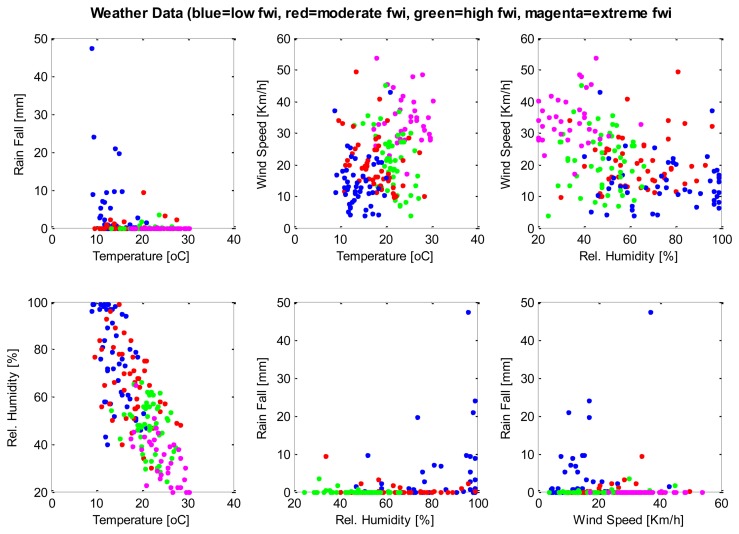
2D classification of the weather database.

**Figure 3.**
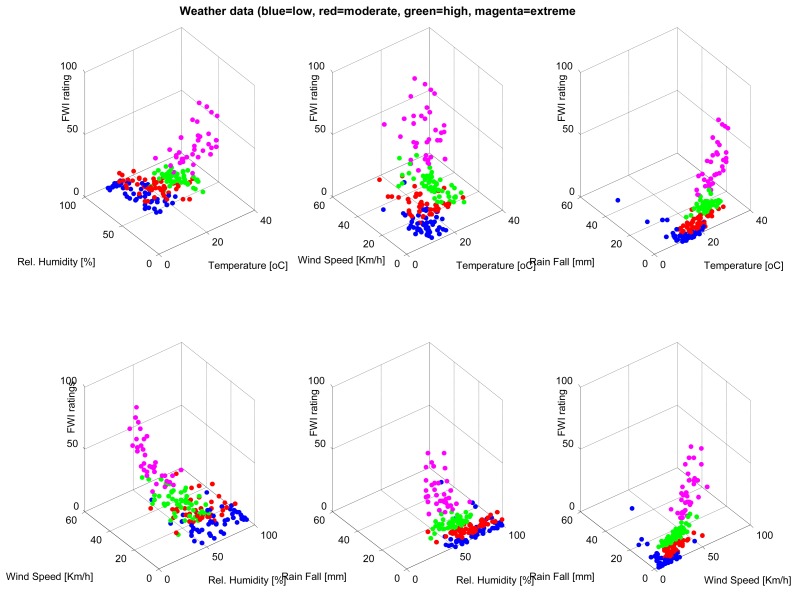
3D classification of the weather database.

A database consisting of 200 instances, each containing weather parameters, are clustered using the SUBFCM algorithm. Each instance of the database has its classification of the fire weather index (FWI) rating. There are four FWI ratings in each instance of the database: low, moderate, high and extreme. Each of the FWI ratings present in the database has 50 examples that, in total, summarise the 200 instances of the database. The graphs below (Figures 2 and 3) show the classification of each instance by comparing each pair of attributes present in the database. The SUBFCM algorithm-generated classes are shown in different colors; blue for a low FWI class, red for a moderate FWI class, green for a high FWI class and magenta for an extreme FWI class. The database contains four attributes: temperature, relative humidity, wind speed and rain fall. The different clusters do not seem to be well separated in 2D depictions. However, they are clearly separate clusters in a 3D graph.

## 3. Results and Discussion

The results presented in this section regarding performance evaluation of the application and the network services are based on averages of 10 to 15 simulation runs with realistic parameters obtained from experimental tests. For the purpose of evaluating the system, we consider two parts: the evaluation of sensor cloud data stream mining quality and evaluation of network services quality The dataset that we used contains 10-minute weather observations of 200 days in Sydney, Australia, recorded from June 2011, to January 2012 [27]. Each day is regarded as a data stream, and each stream has 144 points (24 × 60/10). Each data stream instance consists of temperature, relative humidity, wind speed and rainfall. The data streams are known to represent three levels of forest fire danger ratings (low, moderate and high) on the McArthur Fire Danger Index (FDI) scale [28]. Initially, we evaluate the cluster quality of the sensor cloud model using benchmark standard clustering algorithms; the k-means and the FCM. Using the same data stream sets, we vary the stream dimension and stream periods to investigate the nature and the complexity of streams that can be handled by the model. The first set of simulation considers the system performance on different stream dimensionality and stream rates. Stream sets with single to four dimensions are used to investigate the mining performance with respect to the benchmark models. Sources generating streams as slow as every minute to as fast as every second are used to investigate the effect on cluster quality and validity.

### 3.1. One-Dimensional (ID) Stream Results

**Figure 4.**
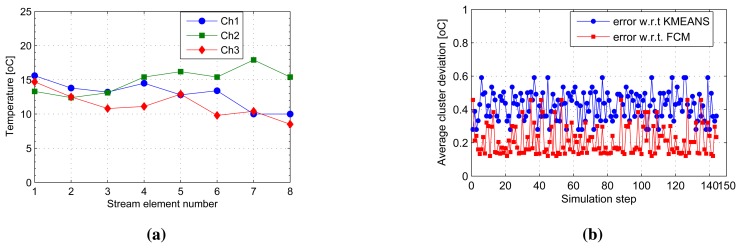
One-dimensional stream results. (**a**) Snapshot of the first values of member nodes; (**b**) average cluster deviations.

Figure 4a shows a snapshot of the first eight elements to have arrived at the cluster heads, Ch1 to Ch3. This makes up the first sliding window of the first three cluster head streams on which the SUBFCM algorithm runs and extracts local cluster models. Analysis of clusters obtained from the SUBFCM system taking into account 144 simulation steps in Figure 4b shows that the temperature cluster centres obtained deviate by 0.4241 °C and 0.2113 °C, on average, in reference to the central k-means and FCM systems, respectively. The maximum cluster centre displacements observed are 0.59 °C and 0.46 °C compared to k-means and FCM systems, respectively. The maximum deviation is only 2.8% of the maximum temperature in the stream.

### 3.2. Two-Dimensional (2D) Stream Results

**Figure 5.**
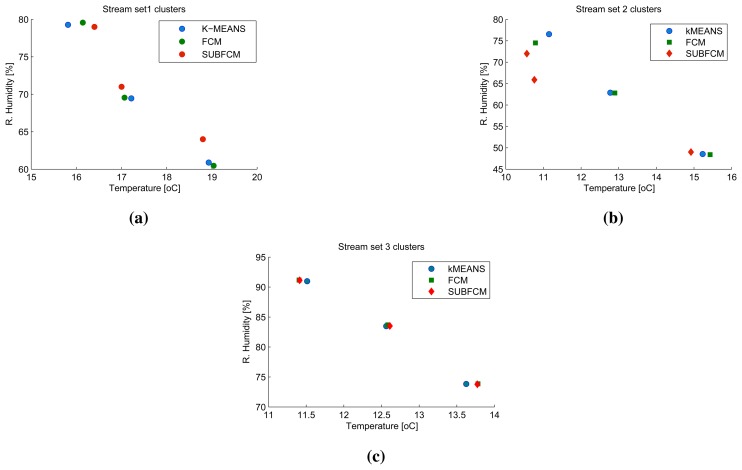
A snapshot of the first sliding windows of cluster heads, one to three. (**a**) snapshot of the first sliding windows of cluster head one; (**b**) snapshot of the first sliding windows of cluster head two; (**c**) snapshot of the first sliding windows of cluster head three.

**Figure 6.**
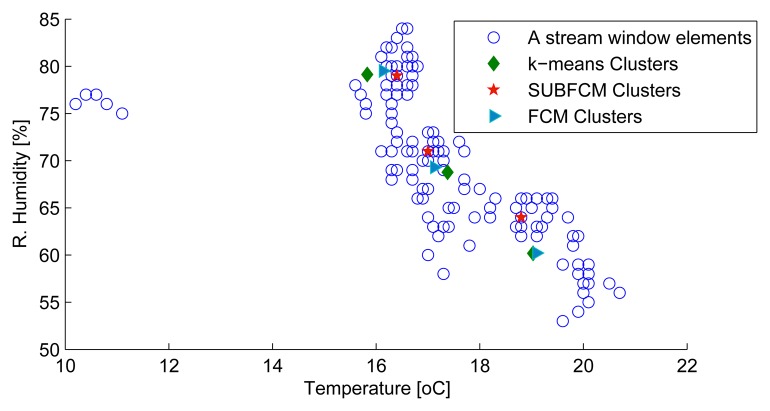
Stream sets and clusters from the first simulation step.

Figure 5a–c shows a snapshot of the first sliding windows of cluster head one to three. The first stream set, taken at the first simulation step, along with the cluster centres obtained using the distributed SUBFCM system and reference systems; k-means and FCM. Analysis of the results obtained shows that the distributed system cluster centres deviate by 3.86% and 1.46% of the cluster radius, on average, with respect to the k-means and FCM cluster centres, respectively. The maximum cluster deviation observed in this simulation is 13.22% with respect to k-means and 5.16% with respect to FCM, as shown in Figure 6.

### 3.3. Three-Dimensional (3D) Stream Results

**Figure 7.**
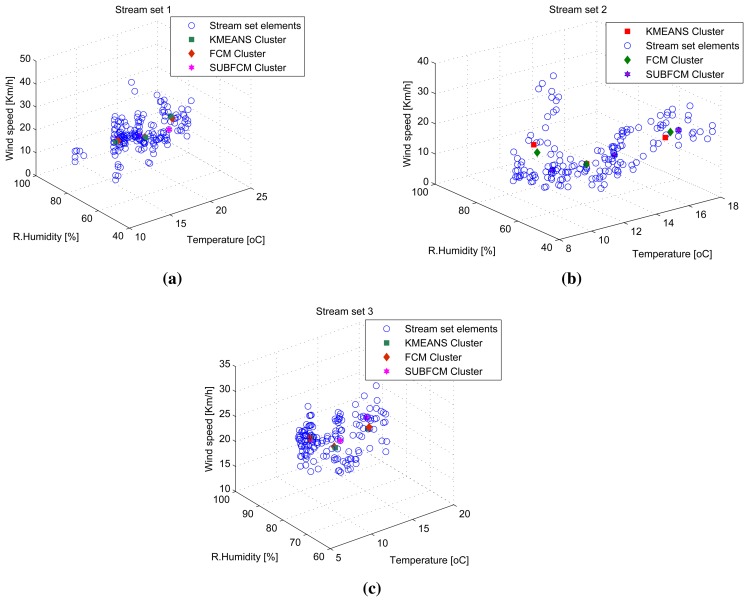
Stream sets and clusters for the first three stream sets. (**a**) Stream sets and clusters for the first stream set; (**b**) stream sets and clusters for the second stream set; (**c**) stream sets and clusters for the third stream set.

**Figure 8.**
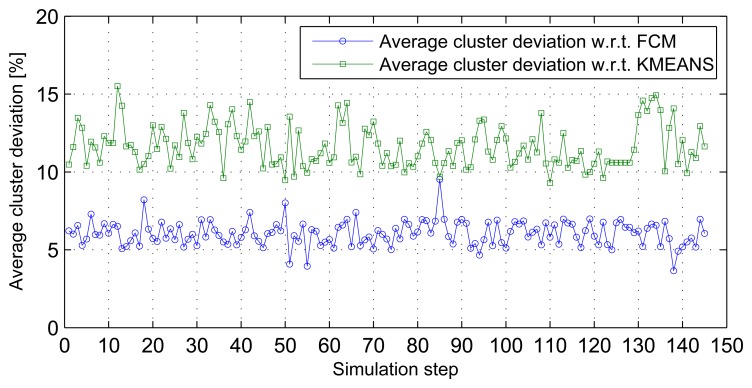
Average cluster deviations with respect to k-means and FCM for 3D streams.

Three-dimensional stream analysis simulation considers three variables (temperature, relative humidity and wind speed) from the same data sources used in previous simulations. Figure 7a-c shows stream sets and clusters for the first three stream sets. Simulation result analysis shows that the average cluster deviations of the distributed SUBFCM system is 11.63% and 6.05% compared to the k-means and FCM systems, respectively. The maximum observed cluster deviation is 15.52% compared to the k-means system. Figure 8 shows average cluster deviations for the 144 simulation runs with respect to k-means and FCM, respectively.

### 3.4. Stream Rate Results

**Figure 9.**
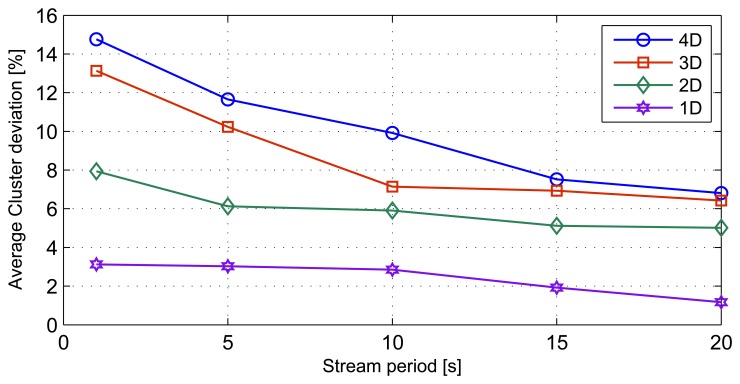
Average cluster deviation variation with stream period.

This simulation involves varying the stream periods. The simulation is then repeated for the different stream dimensions discussed in previous sections. We simulated stream periods of 20 s, 15 s, 10 s, 5 s and 1 s. The effect of stream periods on cluster deviation is shown in Figure 9. The streams with the higher dimensions show higher deviations, as a function of stream period. 4D stream sets show the highest standard deviation of 3.23 around the mean deviation of 10.13%, while ID stream sets exhibit the least standard deviation of 0.85 with a mean cluster deviation of 2.42%. The results indicate that when the data streams consist of higher than 2D elements, the average cluster deviations increase with the increase in the stream periods.

### 3.5. Cluster Density

**Figure 10.**
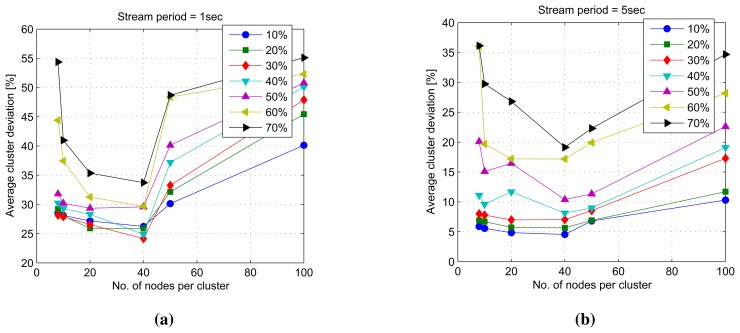
Average cluster deviation with varying cluster densities. (**a**) Average cluster deviation with varying cluster densities in a 1-s stream period; (**b**) average cluster deviation with varying cluster densities in a 5-s stream period.

**Figure 11.**
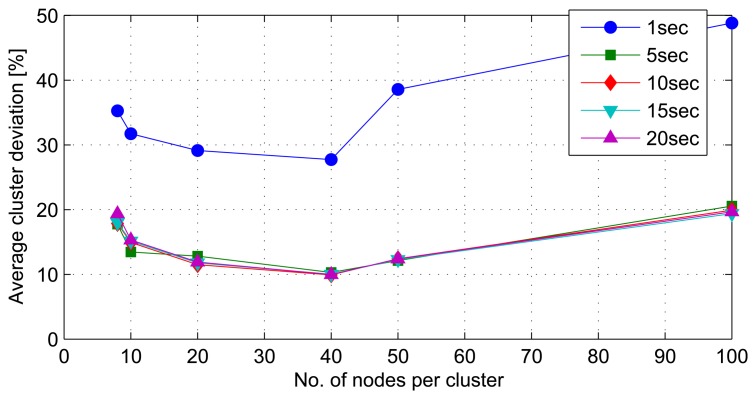
Average of cluster deviations for different stream periods.

The second set of simulations investigates the effect of network architectural variances on the mining performance. The variables considered in this simulation are cluster density, local model drift threshold (*i.e.*, the maximum amount of local model drift before the system starts updating the global model) and non-uniform clusters. Simulations reveal that the optimum cluster density for the best clustering results under the given network architecture is 40 nodes per cluster. The ability of the model to handle data streams arriving in periods of longer than one second is also observed. The stream period of one second or lower is, however, too fast for the model, as manifested in the relatively higher average cluster deviations, as shown in Figures 10 and 11. High cluster deviations at a very low number of nodes per cluster in all simulations point to the fact that by dividing a given large quantity of datasets into smaller sets, mining these smaller sets at distributed locations simultaneously and incrementally extracting the hidden global structures can yield results comparable to that of mining the whole dataset at a central location. However, as the number of divisions increase, the number of distributed mining locations increases with very small sub-sets of data, and the mining results start to degrade in comparison to the central mining results.

### 3.6. Non-Uniform Cluster Density

**Figure 12.**
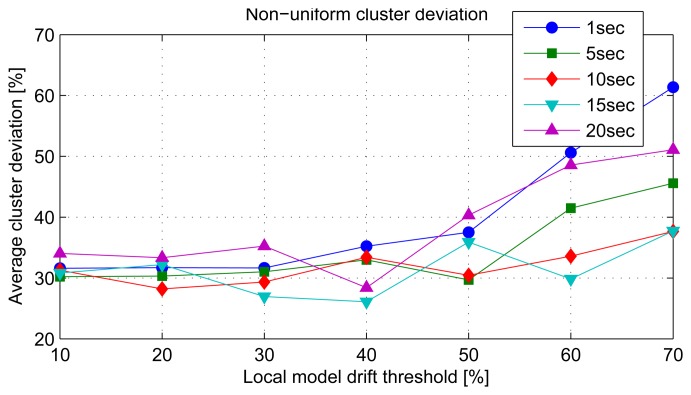
Average cluster deviation for non-uniform cluster density.

For the case of non-uniform cluster density, the average cluster deviation as a function of the local model drift threshold for the different stream periods is plotted in Figure 12. The cluster deviations are generally higher compared to the uniform node densities per cluster. The stream periods exhibit different minimum and maximum cluster deviations for different local model drift thresholds. The irregular cluster deviation pattern is due to the assignment of the same stream period for clusters of different densities.

### 3.7. Network Quality of Service Results

The third and final set of simulation considers the impact of the model by evaluating the network services behavior. This evaluation considers the average energy consumption, the average data delivery delay and the packet delivery ratio.

#### 3.7.1. Average Energy Consumption

**Figure 13.**
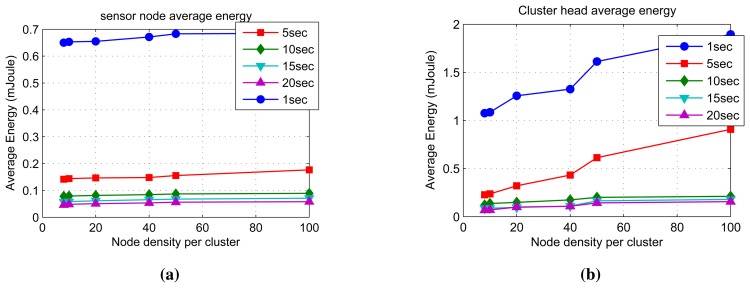
The average energy consumption of the sensor nodes and the cluster heads. (**a**) Sensor node average energy consumption; (**b**) cluster head average energy consumption.

The average energy consumption of a typical node taking the average of all nodes in a cluster for a single step of simulation run is shown in Figure 13a. Similarly, the average energy consumption of a typical cluster head taking the average of all cluster heads in the network for a single simulation step is shown in Figure 13b. The average energy consumption for the entire simulation run can be found by multiplying this with the number of simulation steps. The average energy consumption of the sensor nodes and the cluster heads largely vary with the stream periods and node densities per cluster. The results in Figure 13a show that streams with shorter periods result in higher average energy consumption than those with longer periods. This is because for longer stream periods, the nodes spend more time in sleep mode. The sensor nodes and cluster heads consume above 90% less energy when mining data streams with a period of 20 seconds compared to mining the same data streams with a period of one second. This could also be due to a higher packet collision and lower data delivery ratio at such fast speeds.

#### 3.7.2. Average Data Delivery Delay

**Figure 14.**
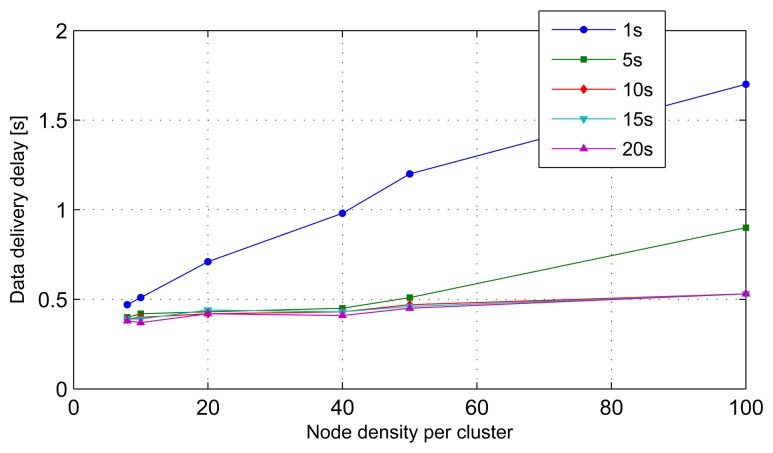
Packet delivery delay variation with cluster density.

The packet delivery delay is defined as the time elapsing between the instant at which a packet is generated at a source and the instant at which a copy of the packet is first received to a destination [29]. In our model, we define the data delivery delay as the time elapsed between the instant a data packet is generated at a source and the instant at which the local cluster models generated at the cluster heads, corresponding to the data packet, arrive at the sink. The average data delivery delay of a typical sensor node is shown in Figure 14. We can observe that the average data delivery delay increases as the number of nodes per cluster increases. For a stream period of one second, the average data delivery delay exceeds the stream period when more than 40 nodes exist per cluster. This situation indicates saturation of the system due to streams arriving at a rate higher than the rate at which the system can process them. However, for stream periods of five seconds or more, the average data delivery delays are well below 500 ms, with the exception of a five-second stream period at more than 50 nodes per cluster density.

#### 3.7.3. Packet Delivery Ratio

**Figure 15.**
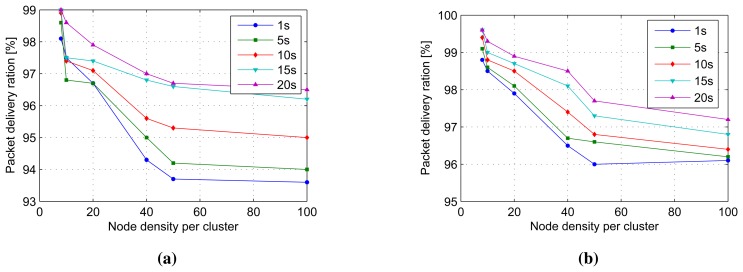
The packet delivery ratios of node-to-cluster head and cluster head-to-sink. (**a**) Packet delivery ratio of sensor node-to-cluster head; (**b**) packet delivery ratio of cluster head-to-sink node.

The packet delivery ratio is the ratio of packets received at the sink to the packets generated by all other nodes [30]. In our model, using cluster architecture and in-network processing, we define two packet delivery ratios; the sensor node-to-cluster head packet delivery ratio and the cluster head-to-sink packet delivery ratio. The former is the ratio of packets received by cluster heads to the packets sent by cluster members; whereas the later is the ratio of packets received by the sink to the packets sent by the cluster head. The packets delivery ratios are observed to vary with stream period and cluster density variations. Packet delivery ratios of between 96.7%–99% are observed when every cluster contains less than 20 nodes, no matter what the stream periods are, as exhibited in Figure 15a. However packet delivery ratios drop rapidly to as low as 93.7% to 96.8% when the node density is increased to 50 nodes per cluster, especially at faster stream periods. The stream periods have a significant impact on the rate of the packet delivery ratio drop as node density increases. This indicates that for optimal performance, applications utilizing the distributed model should limit the cluster density to below 20 if a packet delivery ratio of above 96% is desired. The packet delivery ratios of cluster head-to-sink are shown in Figure 15b. Packet delivery ratios with a drop of 99.5-96.5 are observed for node densities of eight to 50 nodes per cluster for all stream periods, except for a one-second stream period, which further drops to 96%. The lower packet deliver ratio observed is about 96% for a node density of 100 nodes per cluster. For the case of cluster head-to-sink packet delivery ratio, the stream periods do not show a significant impact on the rate of the packet delivery ratio drops, as in the sensor node-to-cluster head packet delivery ratio drops. Applications that can tolerate stream packet delivery ratios as low as 94% for the sensor node-to-cluster head ratio and 96% for the cluster head-to-sink ratio can deploy as much as 100 nodes per cluster given that the increased energy consumption, and data delivery delays, as a consequence, are acceptable.

## 4. Conclusions

The SUBFCM algorithm is a light unsupervised method for the analysis of data and the formulation of empirical models from the gathered data. It has been shown that a systematic combination of subtractive clustering and fuzzy c-means clustering algorithms, SUBFCM, is a light data clustering algorithm computationally suited for low resource systems, like WSN. The sensor cloud data stream mining WSN model is evaluated through simulations. The robustness of the model to different data stream dimensions and data stream rates is demonstrated through the first set of simulations. Benchmarking on standard mining algorithms, the k-means and the FCM algorithms, we have demonstrated that the model can perform high quality distributed data stream mining tasks, comparable to centralised data stream mining. The second set of simulations have shed light on the network architectural design guidelines required to satisfy desirable application demands without compromising the distributed data stream mining task integrity. The third and final set of simulations has also discussed the energy cost and network quality of service impacts for optimal system performance.
